# Influence of abutment materials on peri-implant tissues health—a systematic review

**DOI:** 10.2340/biid.v13.46210

**Published:** 2026-07-02

**Authors:** Waad Kheder, Renita Rego, Marwan Mansoor Mohammed, Zaid Hamdoon, Waseem Jerjes

**Affiliations:** a College of Dental Medicine, University of Sharjah, Sharjah, United Arab Emirates; b College of Dentistry, Al Bayan University, Baghdad, Iraq; c Faculty of Medicine, Imperial College London, London, UK

**Keywords:** Abutments, Titanium, Zirconia

## Abstract

**Background:**

Abutment materials play a critical role in maintaining peri-implant tissue health, influencing both biological responses and aesthetic outcomes in implant-supported restorations. With the introduction of newer materials and digital fabrication techniques, an updated evaluation of their clinical performance is warranted.

**Objective:**

This systematic review aimed to evaluate the influence of different abutment materials—titanium, zirconia, and polyether ether ketone (PEEK)—on peri-implant tissue health based on contemporary clinical evidence.

**Materials and Methods:**

A systematic literature search was conducted in PubMed and Scopus databases in accordance with Preferred Reporting Items for Systematic reviews and Meta-Analyses (PRISMA) guidelines. Clinical studies published between 2019 and 2026 were included to reflect current advancements in implant prosthodontics. Eligible human studies assessing the impact of abutment materials on peri-implant tissue health were selected based on predefined criteria. Risk of bias was assessed using the ROBINS-I tool. Due to heterogeneity in study design, outcome measures, and follow-up duration, a meta-analysis was not feasible, and a qualitative synthesis was performed.

**Results:**

A total of 13 studies were included. Titanium abutments demonstrated consistent performance, with stable marginal bone levels, low inflammatory response and high long-term predictability. Zirconia abutments showed comparable biological outcomes, with superior soft tissue response and aesthetic integration, particularly in anterior regions. Evidence regarding PEEK abutments was limited; however, preliminary findings suggest reduced inflammatory response and favorable biocompatibility. The included studies exhibited substantial heterogeneity in methodology and outcome reporting, limiting direct comparisons.

**Conclusions:**

Titanium remains the most reliable abutment material due to its well-established clinical performance and mechanical stability. Zirconia represents a suitable alternative in aesthetically demanding situations, offering favorable soft tissue outcomes. PEEK demonstrates promising biological potential but lacks sufficient long-term clinical evidence to support routine use. Future research should focus on well-designed, long-term comparative studies with standardized outcome measures to strengthen the evidence base and guide clinical decision-making.

KEY MESSAGES

1. Titanium abutments stay the gold standard, proving superior predictable peri-implant tissue outcomes and mechanical stability.
2. Zirconia abutments offer comparable biological compatibility to titanium and enhanced aesthetic results, supporting their use in esthetically demanding regions.
3. PEEK abutments show promising reduced inflammatory response and biocompatibility; however, long-term comparative studies are warranted, and evidence is limited.

## Introduction

Dental implant therapy has become a well-established and predictable treatment modality for the rehabilitation of partially and completely edentulous patients, offering high survival rates and improved functional and aesthetic outcomes [1]. The long-term success of implant-supported restorations depends not only on osseointegration, but also on the maintenance of healthy peri-implant tissues, which serve as a biological seal protecting the underlying bone from microbial and mechanical challenges [2]. The peri-implant soft tissue complex, consisting of keratinized mucosa and connective tissue, plays a critical role in preserving peri-implant tissue health. Disruption of this interface may lead to biological complications such as peri-implant mucositis and peri-implantitis, which are among the leading causes of implant failure [2]. Consequently, factors influencing the integrity of the peri-implant soft tissue seal have become a major focus in contemporary implant dentistry [3].

Among these factors, the choice of abutment material has received considerable attention. The abutment represents the transmucosal component connecting the implant fixture to the prosthetic restoration and directly interacts with the peri-implant soft tissues. Therefore, its material composition, surface characteristics, and mechanical properties can significantly influence tissue response, bacterial adhesion, inflammatory processes, and marginal bone stability [4]. Titanium has long been regarded as the gold standard abutment material due to its excellent biocompatibility, corrosion resistance, and mechanical strength. Numerous clinical studies have demonstrated its ability to maintain stable peri-implant bone levels and favorable tissue conditions over time [5, 6]. However, aesthetic limitations, particularly in the anterior region, have prompted the development of alternative materials [7].

Zirconia has emerged as a widely used alternative due to its tooth-like color, favorable optical properties, and high biocompatibility [5]. In addition to its aesthetic advantages, zirconia has been associated with reduced bacterial adhesion and improved soft tissue response compared to metallic abutments [6]. Long-term clinical studies have also demonstrated comparable survival rates to titanium, supporting its use in implant prosthodontics [9, 10]. Nevertheless, concerns remain regarding its mechanical behavior and susceptibility to technical complications under functional loading [11]. More recently, polyether ether ketone (PEEK) has been introduced as a novel biomaterial in implant prosthodontics. PEEK is a high-performance polymer characterized by favorable mechanical properties, chemical stability, and potential for reduced inflammatory response [1]. Preliminary clinical evidence suggests that PEEK may support favorable soft tissue integration and reduced inflammatory markers compared to conventional materials [4]. However, its clinical application remains limited, and long-term data are lacking.

Despite the growing body of literature on abutment materials, the available evidence remains heterogeneous, with variations in study design, outcome measures, and follow-up duration. Furthermore, rapid advancements in digital workflows and Computer-Aided Design/Computer-Aided Manufacturing (CAD/CAM) fabrication techniques have introduced new material configurations and surface modifications, necessitating updated evaluations of their clinical performance [3]. Therefore, a comprehensive and up-to-date synthesis of the available clinical evidence is required to better understand the impact of different abutment materials on peri-implant tissue health. This systematic review aims to evaluate and compare the biological and clinical outcomes associated with titanium, zirconia, and PEEK abutments, with a particular focus on peri-implant tissue health parameters, including marginal bone loss, soft tissue response, and inflammatory outcomes.

## Materials and methods

### Study design and registration

This systematic review was conducted in accordance with the Preferred Reporting Items for Systematic Reviews and Meta-Analyses (PRISMA) guidelines [8]. The review protocol was prospectively registered in PROSPERO (CRD420251024493).

### Search strategy

A comprehensive literature search was conducted using PubMed and Scopus databases to identify relevant studies evaluating the influence of abutment materials on peri-implant tissue health. These databases were selected due to their extensive coverage of biomedical and dental literature and their widespread use in implant dentistry research. Although additional databases such as Embase and Cochrane CENTRAL are recognized for their relevance in systematic reviews, they were not included in the present study. This decision was based on feasibility considerations and the expectation that the majority of relevant clinical studies would be indexed within PubMed and Scopus. However, the exclusion of these databases may have limited the comprehensiveness of the search and is acknowledged as a potential limitation.

The search was limited to studies published between January 2019 and December 2026 to capture contemporary clinical evidence reflecting recent advancements in abutment materials, including CAD/CAM zirconia systems and emerging materials such as PEEK. The search strategy included combinations of keywords related to dental implant abutments, abutment materials (titanium, zirconia, and PEEK), peri-implant tissue health, marginal bone loss, inflammation, and soft tissue response. Full search strategies are provided in Table 1.

**Table 1:** Search terms used for PubMed and Scopus databases (from 2004 to 2026).

Database	Search terms	Hits
PubMed	implant abutment material, peri-implant tissue health, abutment surface modifications, abutment morphology, peri-implantitis, one-piece abutments, peri-implant inflammatory response, abutment microgap and bone loss, peri-implant bone stability, microbial colonization around implants	2,880
Scopus	implant abutment material, peri-implant tissue health, abutment surface modifications, abutment morphology, peri-implantitis, one-piece abutments, peri-implant inflammatory response, abutment microgap and bone loss, peri-implant bone stability, microbial colonization around implants	2,482

implant abutment material AND peri-implant tissue health OR peri-implantitis

implant abutment material AND peri-implant inflammatory response OR peri-implant bone stability

implant abutment material AND microbial colonization around implants

abutment surface modifications AND microbial colonization around implants **OR** peri-implant inflammatory response

abutment surface modifications AND peri-implant bone stability OR peri-implant tissue health

abutment surface modifications AND peri-implantitis

abutment morphology AND peri-implant inflammatory response

abutment morphology AND peri-implant tissue health OR microbial colonization around implants

abutment morphology AND peri-implantitis OR peri-implant bone stability

one-piece abutments AND peri-implant tissue health OR peri-implantitis

one-piece abutments AND peri-implant inflammatory response OR peri-implant bone stability

one-piece abutments AND microbial colonization around implants

abutment microgap and bone loss AND microbial colonization around implants **OR** peri-implant inflammatory response

abutment microgap and bone loss AND peri-implant bone stability OR peri-implant tissue health

abutment microgap and bone loss AND peri-implantitis

### Eligibility criteria

#### Inclusion criteria

- Human clinical studies (randomized controlled trials, prospective and retrospective studies, and case series)
- Studies evaluating titanium, zirconia, or PEEK abutments
- Studies reporting outcomes related to peri-implant tissue health (e.g., marginal bone loss, inflammation, soft tissue response, and survival rate)
- English-language publications
- Studies published between 2019 and 2026

#### Exclusion criteria

- Animal or in vitro studies
- Case reports, reviews, and conference abstracts
- Non-English studies
- Studies lacking relevant clinical outcomes
- Studies published prior to 2019

The inclusion of heterogeneous study designs was considered necessary due to the limited availability of high-level clinical evidence, particularly for emerging materials such as PEEK.

### Study selection

**Figure 1:**
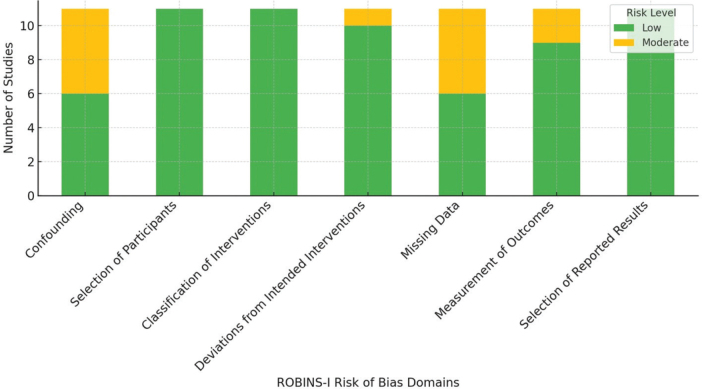
PRISMA flowchart of selection process.

After database searching, duplicates were removed. Titles and abstracts were screened, followed by full-text assessment of eligible studies. The selection process was performed systematically based on predefined criteria and is illustrated in the PRISMA flow diagram (Figure 1).

### Data extraction

Data was extracted using a standardized form including:

- Study design
- Number of patients and implants/restorations
- Abutment material
- Type of restoration
- Follow-up duration
- Clinical outcomes related to peri-implant tissue health

### Risk of bias assessment

Risk of bias was assessed using the ROBINS-I tool across multiple domains. Studies were categorized as low or moderate risk of bias (Table 3).

### Data synthesis

Due to substantial heterogeneity among the included studies in terms of study design, sample size, follow-up duration, outcome measures, and prosthetic protocols, a meta-analysis was not feasible. Therefore, a qualitative synthesis of the findings was performed. The results were analyzed descriptively, focusing on identifying trends and comparative outcomes associated with different abutment materials.

## Results

### Study selection

The initial search of PubMed and Scopus databases identified a total of 5,362 records. After removal of duplicates using automated tools, 158 records remained. Following title and abstract screening, 93 studies were considered potentially eligible. Of these, 28 articles were excluded due to lack of accessible full text. The remaining 65 full-text articles were assessed for eligibility, resulting in the exclusion of 52 studies that did not meet the inclusion criteria. Ultimately, 13 studies were included in the qualitative synthesis (Table 2). The study selection process is illustrated in the PRISMA flow diagram (Figure 1), ensuring transparency and reproducibility.

**Table 2:** Data extraction form shows the characteristics, abutment materials, and results of 11 studies included in the current review.

Author(s)	Year	Aim of the study	Study design/ population	Abutment material	Results (health of peri-implant tissue)	Number of implants	Follow-up duration	Type of restoration
Bernabeu-Mira et al. [14]	2021	To assess the impact of abutment characteristics on marginal bone levels in immediate-loading full-arch prostheses.	Retrospective case series study with 17 patients having full-arch immediate-loading prostheses.	Titanium, Zirconia	Zirconia abutments exhibited less marginal bone loss compared to titanium abutments.	87	1 year	Not mentioned
Donker et al. [2]	2022	To evaluate monolithic zirconia restorations with CAD/CAM titanium abutments in the posterior region.	Prospective case series study with 45 patients having screw-retained monolithic zirconia implant-supported restorations with CAD/CAM titanium abutments in the posterior region.	Titanium	Good clinical, radiographic and patient-reported outcomes for screw-retained monolithic zirconia restorations with CAD/CAM titanium abutments in the posterior maxilla and mandible after 1 year in function.	49	1 year	Zirconia
Enkling et al. [1]	2022	To investigate soft tissue responses to different abutment materials in a controlled model.	Controlled and randomized study with 20 participants where 40 experimental one-piece healing abutment of four different materials were mounted on bone-level implants (split-mouth design).	Titanium, Zirconia	Zirconia abutments demonstrated enhanced soft tissue integration when compared to titanium.	40	3 months	Not mentioned
Fonseca et al. [3]	2021	To assess the clinical and esthetic outcomes as well as patient satisfaction of screw-retained one-piece implant crowns fabricated with zirconia abutments.	Prospective cohort study with 32 patients receiving 40 implant single crowns in anterior and premolar sites.	Zirconia	Zirconia abutments achieved favorable aesthetic outcomes with high survival rates and minimal soft tissue complications.	40	4.5 to 8 years	Zirconia
Hosseini et al. [9]	2024	To assess the survival rate of zirconia vs. metal abutments in patients with agenesis.	Prospective clinical study with 53 patients participated to previously published prospective clinical study with 3-year follow-up were recalled after 5 years.	Zirconia, Metal	Although zirconia and metal abutments exhibited similar survival rates, zirconia yielded superior aesthetic outcomes.	89	5 years	50 zirconia abutments got 50 all-ceramic (AC) crowns, 39 metal abutments got 29 metal-ceramic (MC) and 10 AC crowns.
Koller et al. [11]	2020	To compare clinical outcomes of two-piece zirconia vs. titanium implants.	Prospective randomized pilot trial with 21 patients undergoing posterior restorations.	Titanium, Zirconia	Two-piece zirconia abutments resulted in marginal bone loss comparable to titanium but were associated with improved aesthetics.	28	80.9 months	Not mentioned
Milinkovic et al. [4]	2022	To analyze soft tissue response to PEEK and titanium healing abutments.	Randomized pilot clinical study with 10 patients using PEEK and titanium healing abutments.	PEEK, Titanium	PEEK abutments showed encouraging biocompatibility and elicited fewer inflammatory markers than titanium.	20	3 months	Not mentioned
Pozzi et al. [10]	2023	To evaluate the long-term survival of zirconia screw-retained implant prostheses.	Retrospective multicenter study with 98 patients receiving screw-retained prostheses.	Zirconia	Zirconia-based screw-retained implant-supported prosthesis can be considered a reliable long-term treatment option for partial and complete edentulism.	111	Mean: 7.2 ± 3.4 years	Zirconia
Smirani et al. [7]	2024	Clinical outcomes of single implant-supported crowns utilizing titanium base abutments.	Prospective cohort study with 18 patients receiving single-crown restorations.	Titanium	Titanium abutments ensured long-term stability of peri-implant tissues with minimal marginal bone loss.	18	7.5 years	Zirconia
Strauss et al. [6]	2022	To assess radiographic, restorative, clinical and technical outcomes as well as patient satisfaction of directly veneered zirconia restorations cemented on non-original titanium bases	Prospective cohort study with 22 patients using zirconia restorations on titanium bases.	Zirconia, Titanium	The restorative angle of implant-supported crowns on non-original titanium bases influences the initial marginal bone loss but without affecting their favorable long-term clinical performance.	22	5 years	Zirconia
Weigl et al. [5]	2019	Compare outcomes of all-ceramic vs. titanium-based implant restorations.	A randomized controlled study where 42 patients received single implants restored either by all-ceramic restorations or conventional titanium-based restorations.	Zirconia, Titanium	Zirconia abutments outperformed titanium in aesthetic outcomes and peri-implant tissue health.	42	1 year	21 zirconia abutments got all-ceramic crowns, 21 titanium abutments got PFM crowns
Kumar et al. [12]	2024	To compare the effects of zirconia and titanium abutments on peri-implant hard and soft tissues.	A prospective clinical study included 40 patients requiring single-tooth implant restorations in the posterior region.	Zirconia, Titanium	Zirconia abutments are associated with better peri-implant tissue health and reduced marginal bone loss.	40	6 months	Not mentioned
Savitha et al. [13]	2024	To compare the impact of titanium, zirconia, and gold alloy abutments on peri-implant tissue health and esthetic outcomes in patients receiving implant-supported prostheses.	A randomized controlled trial with 90 patients requiring single-tooth implant-supported prostheses.	Zirconia, Titanium, Gold	Zirconia abutments resulted in the most favorable peri-implant tissue health, with significantly lower PD, BOP, and plaque index compared to titanium and gold alloy abutments. In terms of esthetic outcomes, zirconia abutments also outperformed the other two abutments.	90	1 year	All-ceramic crowns

CAD: Computer-Aided Design; CAM: Computer-Aided Manufacturing; PEEK: Polyetheretherketone; PFM: Porcelain Fused to Metal; PD: Periodontal Pocket; BOP: Bleeding on Probing

**Table 3:** Risk of bias for the included studies using the ROBINS-I (Risk of Bias in Non-Randomized Studies of Interventions) tool.

Study	Bias due to confounding	Bias in selection of participants	Bias in classification of interventions	Bias due to deviations from intended interventions	Bias due to missing data	Bias in measurement of outcomes	Bias in selection of reported results	Overall, bias risk
Bernabeu-Mira et al. [16]	Moderate	Low	Low	Low	Moderate	Low	Low	Moderate
Donker et al. [2]	Low	Low	Low	Low	Low	Low	Low	Low
Enkling et al. [1]	Low	Low	Low	Moderate	Low	Low	Low	Low
Fonseca et al. [3]	Moderate	Low	Low	Low	Low	Moderate	Low	Moderate
Hosseini et al. [9]	Moderate	Low	Low	Low	Moderate	Moderate	Low	Moderate
Koller et al. [11]	Low	Low	Low	Low	Low	Low	Low	Low
Milinkovic et al. [4]	Moderate	Low	Moderate	Low	Low	Low	Low	Moderate
Pozzi et al. [10]	Low	Low	Low	Moderate	Low	Low	Low	Low
Smirani et al. [7]	Moderate	Low	Low	Low	Moderate	Low	Low	Moderate
Strauss et al. [6]	Low	Low	Low	Low	Low	Low	Low	Low
Weigl et al. [5]	Moderate	Low	Low	Low	Low	Low	Low	Moderate
Kumar et al. [12]	Low	Low	Low	Low	Low	Low	Low	Low
Savitha et al. [13]	Low	Low	Low	Low	Low	Low	Low	Low

### Study characteristics

The included studies comprised a range of clinical designs, including randomized controlled trials, prospective cohort studies, retrospective analyses, and case series. Sample sizes varied considerably across studies, reflecting differences in study design and clinical settings. The number of patients and implants/restorations, types of abutment materials, prosthetic designs, and follow-up durations are summarized in Table 2. Titanium and zirconia abutments were the most frequently investigated materials, while evidence regarding PEEK abutments was limited. Follow-up durations ranged from short-term evaluations (approximately 1 year) to long-term observations extending up to 12 years.

### Outcomes according to abutment material

#### Titanium abutments

Titanium abutments were consistently associated with stable peri-implant tissue conditions. Across multiple studies, titanium demonstrated minimal marginal bone loss, low inflammatory response, and high long-term survival rates. These findings reinforce its established role as a reliable material in implant prosthodontics, particularly in load-bearing regions.

#### Zirconia abutments

Zirconia abutments exhibited favorable outcomes in terms of peri-implant tissue health, particularly in soft tissue response and aesthetic integration. Several studies reported improved mucosal conditions and comparable marginal bone stability relative to titanium. Long-term studies also demonstrated satisfactory survival rates; however, occasional technical complications, such as veneering fractures, were noted.

#### PEEK abutments

Evidence regarding PEEK abutments was limited to a small number of studies. Available data suggest that PEEK may be associated with reduced inflammatory response and favorable soft tissue behavior. However, due to the lack of long-term clinical data and limited sample sizes, the evidence remains preliminary.

### Comparative analysis

**Figure 2:**
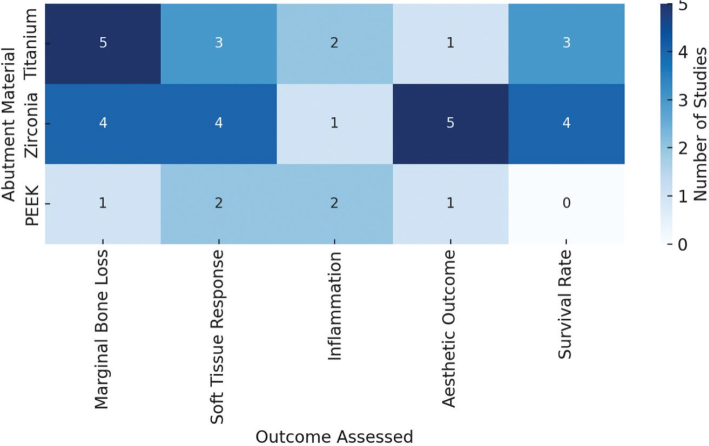
Evidence Map: Outcome assessed by Abutment Material. This matrix summarizes the number of included studies (*n* = 13) evaluating key clinical outcomes—marginal bone loss, soft tissue response, inflammation, aesthetic outcomes, and survival rate—across different abutment materials (titanium, zirconia, and PEEK). Darker shading indicates a greater number of studies assessing each outcome for a given material. The figure demonstrates a relatively strong and balanced evidence base for titanium and zirconia across most outcome domains, while highlighting the limited and emerging evidence for PEEK, particularly with respect to long-term survival.

**Figure 3:**
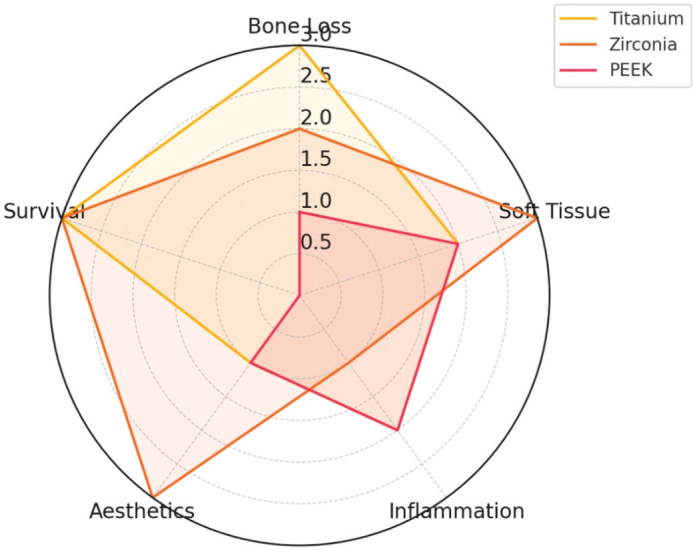
Outcome Advantage Profile per Abutment Material. This radar chart illustrates the relative performance of titanium, zirconia, and PEEK abutments across five clinical outcome domains: marginal bone loss, soft tissue response, inflammation, aesthetic outcomes, and survival rate. Scores (0–3) were assigned based on the strength and frequency of favorable outcomes reported in the included studies. Titanium and zirconia demonstrate comprehensive performance profiles, with distinct advantages in mechanical and aesthetic domains, respectively. In contrast, PEEK shows emerging potential but remains limited by insufficient evidence, particularly regarding long-term survival.

Overall, titanium and zirconia abutments demonstrated comparable biological performance in maintaining peri-implant tissue health, with distinct advantages in mechanical and aesthetic domains, respectively. Titanium showed superior mechanical stability and long-term predictability, whereas zirconia provided enhanced aesthetic outcomes and soft tissue integration. In contrast, PEEK showed emerging potential but lacked sufficient evidence to support direct comparison with established materials. Graphical representations (Figures 2 and 3) illustrate the distribution of outcomes across abutment materials, highlighting the relatively balanced evidence base for titanium and zirconia and the limited data available for PEEK.

### Risk of bias assessment

The methodological quality of the included studies was assessed using the ROBINS-I tool. Most studies were classified as having low to moderate risk of bias across the evaluated domains. The most common sources of bias were related to confounding factors and incomplete data reporting.

**Figure 4:**
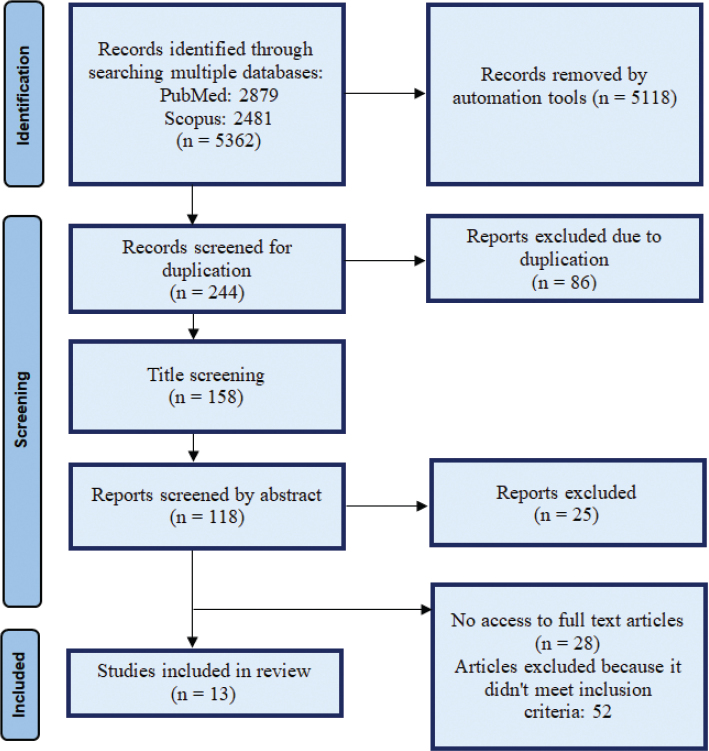
ROBINS-I Risk of Bias Across Domains in Included Studies. This stacked bar chart presents the distribution of included studies (*n* = 13) categorized as having low or moderate risk of bias across the seven domains assessed using the ROBINS-I tool. Most studies demonstrated a low risk of bias in domains such as participant selection, classification of interventions, and outcome measurement/reporting. In contrast, the highest proportion of moderate risk was observed in the confounding and missing data domains, highlighting potential limitations related to study design and data completeness.

The distribution of risk of bias across studies is presented in Figure 4, which demonstrates that the majority of studies showed low risk in domains such as participant selection and outcome measurement, while moderate risk was more frequently observed in confounding and missing data domains.

### Summary of findings

The collective evidence indicates that titanium remains the most extensively studied and clinically reliable abutment material, while zirconia offers comparable biological outcomes with additional aesthetic benefits. PEEK represents a promising alternative; however, current evidence is insufficient to draw definitive conclusions regarding its long-term clinical performance.

## Discussion

This systematic review evaluated the influence of abutment materials—titanium, zirconia, and PEEK—on peri-implant tissue health based on contemporary clinical evidence. The findings indicate that titanium remains the most predictable material in terms of mechanical stability and maintenance of marginal bone levels, whereas zirconia demonstrates superior aesthetic outcomes and favorable soft tissue response. PEEK, as an emerging material, shows promising biological behavior; however, the current evidence base remains limited and predominantly short term. Titanium abutments continue to be regarded as the gold standard due to their well-documented biocompatibility, mechanical strength, and long-term clinical success. Across the included studies, titanium consistently exhibited minimal marginal bone loss and stable peri-implant tissue conditions, supporting its continued use, particularly in posterior regions where functional demands are high [2, 6]. These findings are consistent with previous clinical studies reporting stable peri-implant bone levels and favorable biological outcomes associated with titanium abutments [6, 7].

In contrast, zirconia abutments showed comparable survival rates while offering enhanced aesthetic integration and improved soft tissue outcomes. Several included studies demonstrated improved mucosal conditions and favorable biological responses when compared to titanium [5, 9]. Long-term investigations have further supported the clinical reliability of zirconia, reporting stable outcomes over extended follow-up periods [9, 10]. These findings are particularly relevant in anterior regions, where aesthetic considerations are critical. Kumar et al. reported that zirconia abutments were associated with improved soft tissue parameters and better preservation of marginal bone levels compared with titanium abutments over a 6-month observation period. The favorable clinical performance of zirconia may be related to its smoother surface characteristics and lower bacterial affinity, which may reduce plaque accumulation and inflammatory responses around implants [12]. Recent clinical evidence demonstrates that zirconia abutments are associated with reduced probing depth and lower bleeding on probing compared to titanium, indicating decreased peri-implant inflammation [13]. However, some studies reported technical complications associated with zirconia, such as veneering fractures or prosthetic issues, which may influence long-term performance [11]. Therefore, careful case selection remains essential when considering zirconia as an alternative to titanium.

The evidence regarding PEEK abutment remains limited, with only a small number of clinical studies available. Preliminary findings suggest that PEEK may reduce inflammatory responses and support favorable soft tissue integration, potentially due to its bioinert properties [4]. However, the absence of long-term clinical data and the limited number of comparative studies restrict the ability to draw definitive conclusions. Therefore, PEEK should currently be considered an emerging material with promising potential, warranting further investigation through well-designed longitudinal clinical trials.

A key limitation of this review is the heterogeneity among the studies included. Variations in study design, sample size, follow-up duration, outcome measures, and prosthetic protocols limited direct comparability and precluded the performance of a meta-analysis. This heterogeneity also introduces challenges in synthesizing the evidence and may affect the generalizability of the findings. Similar methodological variability has been reported in previous implant dentistry research, highlighting the need for more standardized clinical study designs [3]. Another important limitation relates to the variability in outcome reporting across studies. Differences in how peri-implant tissue health parameters—such as marginal bone loss, soft tissue response, and inflammatory markers—were defined and measured hinder direct comparisons and data pooling. This highlights the need for standardized outcome measures and reporting protocols in future clinical studies to enhance comparability and facilitate higher-level evidence synthesis⁸.

The present review was also limited by the restriction of the literature search to PubMed and Scopus databases. Although these databases provide extensive coverage of biomedical and dental literature, it is possible that relevant studies indexed in other databases, such as Embase or Cochrane CENTRAL, were not captured. Additionally, the inclusion of studies published only between 2019 and 2026 may have excluded earlier foundational research, thereby reducing the breadth of the evidence base. However, this temporal restriction was intentionally applied to focus on contemporary clinical practices and recent advancements in abutment materials, including CAD/CAM zirconia systems and emerging materials such as PEEK [3].

From a clinical perspective, the findings of this review emphasize the importance of a patient-centered approach in abutment material selection. Titanium remains the material of choice in situations requiring high mechanical strength and long-term reliability, particularly in posterior load-bearing regions. Zirconia offers distinct advantages in aesthetically demanding areas, providing improved soft tissue integration and favorable visual outcomes. While PEEK shows potential as a biologically favorable material, its routine clinical application should be approached cautiously until more robust long-term evidence becomes available [4]. Future research should focus on well-designed prospective clinical trials with larger sample sizes and extended follow-up periods to evaluate the long-term performance of both zirconia and PEEK abutments. Additionally, direct comparative studies using standardized outcome measures are essential to enable meaningful comparisons across materials. The integration of patient-reported outcomes, such as aesthetic satisfaction and quality of life, may also provide valuable insights into clinical decision-making. Establishing standardized reporting frameworks will be critical to advancing the evidence base and supporting more definitive conclusions in future systematic reviews.

## Conclusion

This systematic review highlights the pivotal influence of abutment materials on peri-implant tissue health. Titanium remains the most reliable material, demonstrating superior mechanical stability and minimal marginal bone loss. Zirconia is a suitable alternative, particularly in esthetically demanding regions, providing comparable biological outcomes with enhanced soft tissue integration. Preliminary clinical evidence suggests that PEEK may offer favorable biocompatibility and reduced inflammatory responses; however, long-term clinical data are limited. Selection of abutment materials should be guided by patient-specific functional and aesthetic requirements, with zirconia favored in high-aesthetic zones and titanium preferred where mechanical strength is critical. The adoption of PEEK in routine practice should be approached cautiously until further long-term studies are available. Future research should focus on well-designed trials with larger sample sizes, standardized outcome measures, and extended follow-up to strengthen the evidence base and guide clinical decision-making.

## Data Availability

Data sharing is not applicable to this article as no new data were created or analyzed in this study.

## Future directions

Future research should prioritize well-designed, prospective clinical trials with larger and more diverse patient populations to strengthen the evidence base regarding abutment materials. Specifically, studies should aim for longer follow-up periods to capture late-onset biological or technical complications, particularly for emerging materials such as PEEK. Standardization in outcome reporting—especially in peri-implant bone loss, soft tissue health, and inflammatory markers—would facilitate meaningful comparisons and enable future meta-analyses. There is also a need for interdisciplinary research integrating periodontology, prosthodontics, and materials science to explore the biological interactions between novel abutment surfaces and peri-implant tissues. Investigating patient-centered outcomes such as comfort, satisfaction, and quality of life will provide valuable context alongside clinical metrics. Additionally, comparative cost-effectiveness studies could inform material selection in settings with constrained resources. Finally, future systematic reviews may benefit from incorporating grey literature and non-English sources to mitigate language bias and broaden the global relevance of findings. As implant dentistry continues to evolve, such comprehensive and methodologically rigorous studies will be essential to guide clinicians in tailoring abutment selection to individual patient needs and clinical scenarios.
